# SoundCompass: A Distributed MEMS Microphone Array-Based Sensor for Sound Source Localization

**DOI:** 10.3390/s140201918

**Published:** 2014-01-23

**Authors:** Jelmer Tiete, Federico Domínguez, Bruno da Silva, Laurent Segers, Kris Steenhaut, Abdellah Touhafi

**Affiliations:** 1 Department of Electronics and Informatics (ETRO), Vrije Universiteit Brussel, Pleinlaan 2, Elsene 1050, Belgium; E-Mails: fedoming@vub.ac.be (F.D.); ksteenha@etro.vub.ac.be (K.S.); abdellah.touhafi@vub.ac.be (A.T.); 2 Department of Industrial Sciences and Technology (INDI), Vrije Universiteit Brussel, Pleinlaan 2, Elsene 1050, Belgium; E-Mails: BrunoTiago.Da.Silva.Gomes@ehb.be (B.S.); lasegers@vub.ac.be (L.S.)

**Keywords:** SoundCompass, MEMS microphone, microphone array, beamforming, wireless sensor networks, sound source localization, sound map, noise map

## Abstract

Sound source localization is a well-researched subject with applications ranging from localizing sniper fire in urban battlefields to cataloging wildlife in rural areas. One critical application is the localization of noise pollution sources in urban environments, due to an increasing body of evidence linking noise pollution to adverse effects on human health. Current noise mapping techniques often fail to accurately identify noise pollution sources, because they rely on the interpolation of a limited number of scattered sound sensors. Aiming to produce accurate noise pollution maps, we developed the SoundCompass, a low-cost sound sensor capable of measuring local noise levels and sound field directionality. Our first prototype is composed of a sensor array of 52 Microelectromechanical systems (MEMS) microphones, an inertial measuring unit and a low-power field-programmable gate array (FPGA). This article presents the SoundCompass’s hardware and firmware design together with a data fusion technique that exploits the sensing capabilities of the SoundCompass in a wireless sensor network to localize noise pollution sources. Live tests produced a sound source localization accuracy of a few centimeters in a 25-m^2^ anechoic chamber, while simulation results accurately located up to five broadband sound sources in a 10,000-m^2^ open field.

## 1. Introduction

Sound is everywhere in our daily environment, and its presence or absence has a direct effect on our health and lives. Numerous technologies to measure sound—its loudness, its nature, its source—are therefore found in the environmental, industrial and military domain. In all these domains, localizing sound sources is of vital importance, and applications range from localizing sniper fire to identifying noisy engine parts. With a broad range of sound source localization applications in mind, we developed the SoundCompass, an acoustic sensor capable of measuring sound intensity and directionality.

**Figure 1.**
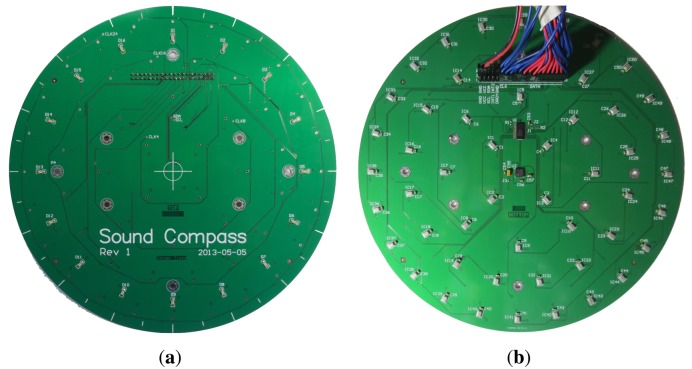
The SoundCompass circuit board without the field-programmable gate array (FPGA) add-on board. (**a**) The top view of the SoundCompass microphone array; (**b**) the bottom view of the SoundCompass microphone array with the debug cable attached.

The SoundCompass, as its name implies, is a compass for a sound field. It points to the direction of the loudest sound sources, while measuring the total sound pressure level (SPL). Our prototype is a 20-cm circular printed circuit board (PCB) (Figure 1) containing a sensor array of 52 microphones, an inertial measurement unit (IMU) and a low-power field-programmable gate array (FPGA). It can work as a standalone sensor or as part of a distributed sensing application in a wireless sensor network (WSN).

The driving application of the SoundCompass is noise pollution mapping in urban environments. Urban noise pollution is of great concern to human health [1,2] and determining human exposure through noise mapping is among the priorities of the European Union environmental policies [3].

**Figure 2.**
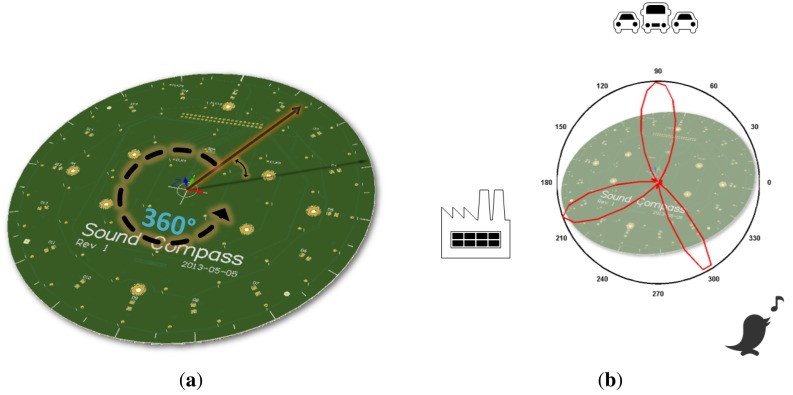
The SoundCompass is a microphone array designed to spatially sample its surrounding sound field. Using beamforming techniques, it performs a 360° sound power scan comprised of a configurable number of discrete angles (**a**). A polar power plot of this scan points to the direction of nearby sound sources (**b**).

The sound field directionality is calculated with the microphone array on board the SoundCompass. Using beamforming, the microphone array measures the relative sound power per horizontal direction to form a 360° overview of its surrounding sound field (Figure 2). The total SPL is measured directly by the sensor’s microphones.

The SoundCompass measures the relative sound power for a discrete number of directions or angles. The number of measurements per 360° sweep is the *angle resolution* of the SoundCompass. These measurements, when represented in polar coordinates, form a *polar power plot*. In non-diffuse sound fields [4], the lobes of this power plot can then be used to estimate the bearing of nearby sound sources (Figure 2b).

The spatial output of a microphone array is commonly known as the steered response power (SRP) [5]. SRP encompasses a broad range of spatial outputs from direction or bearing, similar to the polar power plot described above, to actual three-dimensional positioning. A polar power plot is a type of SRP and, in this article, will be referred to as the polar steered response power or P-SRP.

Several applications could potentially exploit the SoundCompass’s capability to point out the bearing of sound sources as a standalone sensor. However, the SoundCompass has also been designed to function in a distributed manner as part of a WSN. A WSN composed of nodes, each equipped with a SoundCompass, will be able to sample the sound field directionality and SPL in several geographical points. By fusing this information, applications, such as sound source localization and real-time noise maps, are possible.

The SoundCompass is presented here as a standalone sound field sensor. Nevertheless, its design does not overlook the unique constraints found in high-spatial density WSNs, such as low-power consumption and a small size. Additionally, strict timing constraints—sampling rates and global synchronization—are necessary to track mobile and transient sound sources.

This article presents the hardware and firmware design of our prototype together with a data fusion technique that exploits the use of the SoundCompass in a WSN to localize noise pollution sources with the future aim to generate accurate noise maps.

## 2. Related Work

The use of distributed microphone arrays for sound source localization is a well-researched problem that has found applications ranging from sniper localization in urban warfare to wildlife localization in terrestrial environments. The bulk of sound source localization research is found in military applications. For example, Vanderbilt University has been working for several years on developing a counter-sniper system [6]. This system is a WSN solution, where each sensor node, mounted on top of a soldier’s helmet, has a microphone array of four analog microphones connected to an FPGA for signal processing. The system uses domain knowledge (muzzle blast sound signature, typical bullet speed, *etc.*) to locate, via the angle of arrival (AoA) and triangulation, the sniper’s position.

In environmental applications, distributed microphone arrays have been used to monitor wildlife in remote areas [7]. In Collier *et al.*, the authors used a distributed system consisting of eight nodes to monitor antbird populations in the Mexican rainforest [8]. Each node was a VoxNet box, a single-board computer (SBC) equipped with a four-microphone array and capable of storing hours of raw audio data [9]. The nodes were strategically placed in the rainforest to record bird songs. All recorded data had to be manually extracted every day on the field, and sound source localization, using AoA and triangulation, was done offline as a data post-processing task.

Distributed microphone arrays systems have been proposed for indoor applications, such as videoconferencing, home surveillance and patient care. One notable example was the work done by Aarabi, where he demonstrated that sound sources could be located with a maximum accuracy of 8 cm [10]. He used 10 two-microphone arrays distributed in a room and used their data to locate three speakers. However, most of the research that followed this work used either distributed microphones (no arrays) to locate speakers or events (broken windows, falls) within a room [11] or a single microphone array to detect speech in teleconferencing applications [12,13].

Most distributed microphone arrays systems are designed for a specific application domain, where the sound sources of interest are known in advance (e.g., gun shots, birds, speech). In these systems, sound source localization usually follows these steps [10]:

1. Source detection: Correlate the output of one microphone with an expected sound signature or with another microphone using generalized cross-correlation (GCC) [5].
2. Bearing determination: Once correlated, determine the exact bearing of the sound source using time difference of arrival (TDOA).
3. Triangulation: Use the bearings of two or more arrays to triangulate the exact position of the sound source.

Few systems exist in which sound sources are *blindly* detected and located. The most well known and accepted blind sound source localization technique using microphones arrays is the SRP-phase transformation filter (SRP-PHAT), developed by Joseph DiBiase in [5]. He used an array of distributed microphones within a room and integrated their signals to create a room-wide microphone array. The array’s SRP output was used together with a phase transformation filter (PHAT) to counteract the effects of reverberations and accurately locate speakers within a room.

Similarly, Aarabi used the Spatial Likelihood Function (SLF)—a mathematical construction equivalent to SRP—produced by each microphone array and integrated them using a weighted addition to locate all sound sources. He assigned the weights given to each array in accordance to how well positioned they were to detect a sound source [10]. However, most of this research was aimed at speech detection in indoor environments and was therefore adjusted to this application domain.

On the other hand, Wu *et al.* used Blind Source Separation (BSS), the extraction of original independent signals from observed ones with an unknown mixing process, to locate sound sources in an external urban environment [14]. They used three two-microphone arrays to locate two sound sources in a non-reverberant environment and reported a localization error of 10 cm. The microphone arrays were ultimately meant to be ears that could face different directions (the application domain was robotics), and their results required heavy data post-processing to obtain the reported accuracy. A similar method and for the same application domain is proposed by [15], where a microphone array mounted on a robot uses the multiple signal classification algorithm (MUSIC) to detect sound sources in 3D space.

Researchers at the University of Crete are using matching pursuit-based techniques to discriminate multiple sound sources in the time-frequency domain [16]. They tested their techniques using a circular microphone array in a reverberant room environment. This array, composed of eight microphones, estimated the AoA and total number of speakers in a room. However, their application domain is speech localization for videoconferencing applications using a single microphone array; sound source localization is therefore not being performed.

Researchers at the Tallinn University of Technology are investigating the application of SRP-PHAT in a WSN using linear arrays [17]. They have managed to simplify the computational complexity of SRP-PHAT to efficiently use it in a resource constrained environment. However, they are just starting with this work and have published, so far, only preliminary results.

The SoundCompass has been designed to sample the directionality of the sound field of an urban environment where multiple sound sources of different characteristics might be present. Functioning as part of a distributed network of microphone arrays, the directionality data it produces must be fused fast enough to produce a near real-time sound map of an urban area. While our data fusion technique is partly based on the work done by Aarabi and DiBiase, all of the localization techniques and array technologies presented above are too domain specific to be applicable in our domain. Therefore, the SoundCompass differentiates itself from these solutions by using a circular Microelectromechanical systems or MEMS-microphone array combined with an SRP-based data fusion technique to locate sound sources in a new application domain: noise pollution mapping through environmental sensor networks.

## 3. Sensor Architecture

As mentioned in the introduction, the SoundCompass is composed of a microphone array that uses beamforming to measure the directionality of its surrounding sound field. In this section, we will explain the details of how the SoundCompass hardware works and explain the software as used in our prototype.

### 3.1. SoundCompass Hardware

**Figure 3.**
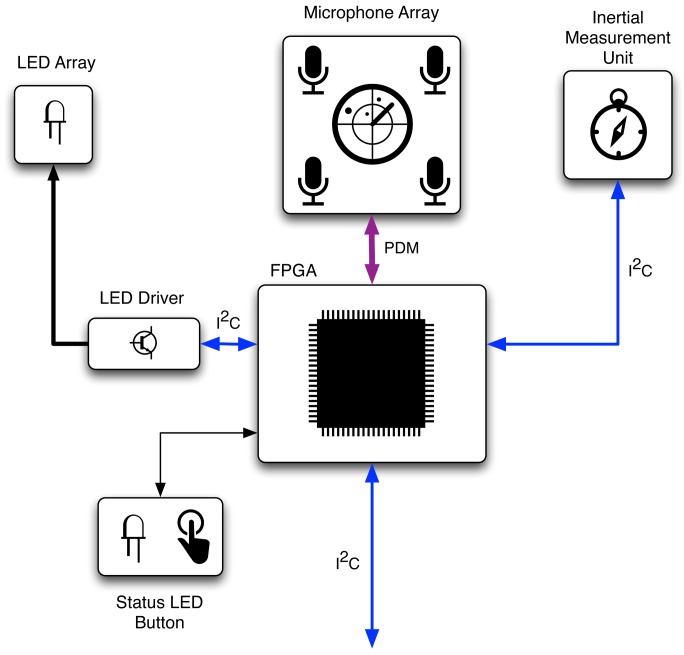
The basic hardware blocks of the SoundCompass. The FPGA is connected via an Inter-Integrated Circuit (I^2^C) interface with the host platform and receives the audio data from the microphone array via a bus of pulse density modulated (PDM) signals.

The SoundCompass consists of an array of digital MEMS microphones mounted in a specific pattern on a circular circuit board with a diameter of 20 cm. An FPGA takes care of the main processing and an IMU determines the orientation of the sensor. The basic hardware blocks of the SoundCompass are shown in Figure 3.

The SoundCompass was designed to work as an individual sensor. This offers us the possibility and flexibility to use the sensor as a standalone module that can be added to any platform or controller. For this reason, we chose to use a simple digital protocol by which engineers can interface the sensor to their preferred platform. Because the amount of data that needs to be transferred is fairly small—one power value per measured angle—we selected the industry standard Inter-Integrated Circuit (I^2^C) interface.

We decided to build the SoundCompass with a combination of digital MEMS microphones and an FPGA instead of analog microphones and a dedicated digital signal processor (DSP). This eases the development, lowers the component count and results in a lower cost of the sensor.

The digital MEMS microphones—off-the-shelf components commonly found in mobile phones—used on the SoundCompass have two main advantages: high value (low-cost/high-quality) and a small package size. These microphones integrate an acoustic transducer, a preamplifier and a sigma-delta converter into a single chip. The digital interface allows easy interfacing with the FPGA without utilizing extra components, such as an analog-to-digital converter, which would be needed for analog microphones. The small package size of these microphones allows for easy handling by a common pick and place machine when assembling the array of the sensor. Microphones designed by Analog Devices (part number: ADMP521) were used in the SoundCompass prototype, because of their fairly good wide-band frequency response from 100 Hz up to 16 kHz and their omnidirectional polar response [18]. These microphones need only a clock signal between one and 3 MHz as the input. The output is a multiplexed pulse density modulated (PDM) signal. The microphones on the SoundCompass prototype currently run at 2 MHz. When no clock signal is provided, the microphones enter a low-power sleep mode (<1 μA), which makes the SoundCompass more suitable for battery powered implementations.

We chose to use an FPGA on the SoundCompass, because all the data originating from the digital MEMS microphones needs to be processed in parallel. Since the sensor will eventually need to be able to operate on battery power in a sensor network environment, we opted for a power efficient FPGA. Our preference went to a flash-based FPGA, mainly because of the absence of the inrush and configuration power components. This enabled the sensor to enter a low power state and conserve battery power, but still wake up fast enough to take a semi-real-time measurement. We selected the Microsemi Igloo series for the SoundCompass prototype [19].

The measurements coming from the SoundCompass will consist of directional power measurements. When multiple sensors are used and measurements are combined and compared, there must be a common knowledge of direction among the different SoundCompasses. One could align all compasses to the same direction while deploying them, but this would result in unreliable measurements if one of the SoundCompasses were moved or even bumped after deployment. For this reason, we chose to add an IMU to the SoundCompass. This sensor enables the compass to determine its own position in space and magnetic north. By normalizing every measurement to magnetic north, we can easily compare measurements originating from different SoundCompasses. Incorporating an IMU on our sensor’s PCB also enables us to detect when a SoundCompass has been moved or repositioned. The IMU selected in our prototype consists of a 3D accelerometer and a 3D magnetometer to form an orientation sensor detecting six degrees of freedom. This enables us to compute a tilt compensated compass [20] and to detect the magnetic north to normalize the measurements taken by a SoundCompass.

We chose a circular PCB for the microphone array itself with an FPGA add-on board on the bottom for modularity reasons. To give visual feedback to a user who is using an individual SoundCompass, a circular array of light emitting diodes (LEDs) is located on top of the microphone array’s PCB. These LEDs are controlled by an I^2^C-led driver. They can give a quick indication of the sound source direction and can be controlled from the FPGA or the host platform through the I^2^C bus. The particular shape of our PCB was determined by the prerequisites we defined for our sensor and is further explained in Section 4.

### 3.2. Sensor Software

**Figure 4.**
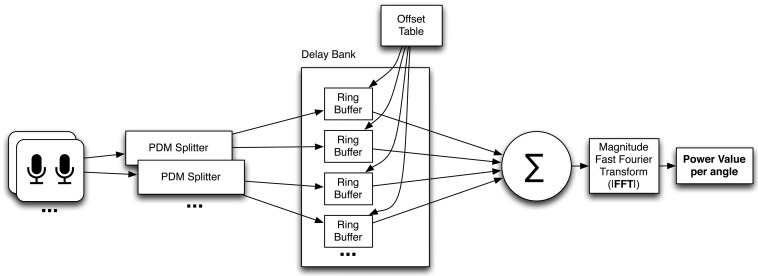
The FPGA structure of the SoundCompass.

The main task of the SoundCompass’s FPGA is to parallel process all digital audio signals coming from the 52 MEMS microphones and to produce a power value for a certain angle or direction. The data streams coming from the microphones are in a one bit-wide pulse density modulated format. This means that the relative density of the pulses corresponds with the amplitude of the analog audio signal. The PDM signal coming from the microphones is multiplexed per microphone pair; this allows us to use one signal line for two microphones. This means that the total bus width and pins used on the FPGA to interface the microphones is equal to half the amount of microphones. To demultiplex these signals, we use the first block in the FPGA structure, which is an interface block that splits the multiplexed PDM signals into 52 separate one bit data streams (Figure 4). This block runs at the same speed as the incoming PDM signal (2 MHz) and consists of 26 sub-blocks that each extract two one-bit data streams from each multiplexed microphone input signal. These sub-blocks extract the signal from the first microphone of the pair on the rising clock edge and the second one on the falling edge.

The next block in the FPGA schematic is the delay bank. This block delays the different digital audio streams for the beamforming algorithm. Each time the SoundCompass focuses on a different angle, the delay values of the different PDM streams are adjusted by the control block in such a way that the focus of the sensor points to the correct direction. The overall size of the delay bank is dependent on the amount of microphones, sampling speed and physical size of the microphone array. Every individual channel in the delay bank has the same width and length. The width is determined by the one bit-wide incoming PDM signal, and the length is determined by the maximum possible delay for all microphones. The calculation of the right delay for every individual microphone is further explained in Section 5.2.2. and Figure 15a. In the case of the SoundCompass and its 52 microphones operating at 2 MHz in a circular layout with a maximum diameter of 18 cm, the amount of storage needed for the maximum delay is 1,048 bits. This results in a delay bank size of 52 × 1,048 bits ≈ 7 kB. The microphone data streaming into the delay bank is written to the current write pointer address, which is the same for every channel and is advanced by one every clock cycle. The read pointer follows the write pointer at a delay distance calculated by the offset table based on the current focus angle. Both pointers wrap around to form a ring buffer.

After exiting the delay bank, all the individual PDM signals are summed together. This sum generates a signal with digital values ranging from zero to the amount of microphones in the array. With 52 microphones, the output signal of this sum has a maximum bit width of six bits. This summed signal is then fed into a fast Fourier transform (FFT) block. The FFT of the 2 MHz signal gives us a usable power spectrum from zero till 1 MHz. Since we are only interested in the audible frequencies from 20 Hz until 20 kHz, we discard all the frequency bins that are not part of this interval. To get a power spectrum in this resulting interval with a high enough resolution, we need a high enough amount of data points for the FFT. This is why we chose to take a 2^16^-point FFT of the summed microphone data stream. After zero-padding and windowing the incoming data points, the FFT block results in a power value, per frequency band, for the incoming audio from the selected direction.

A timing block implements all the timing circuitry to drive the microphones and delay bank, while a control block changes the configuration of the delay bank depending on the focus direction of the microphone array. The total sensing time for one measurement of the SoundCompass, which entails a 360° sweep of the surrounding sound field, depends on the resolution chosen for the measurement. This is further explained in Section 5.2.2. A separate I^2^C controller block handles all communications with the IMU and host platform. This host platform can configure the SoundCompass and request a measurement. When the measurement is complete, the SoundCompass will set a flag that allows the host platform to read out the results from predefined registers.

## 4. The SoundCompass Array Geometry

**Figure 5.**
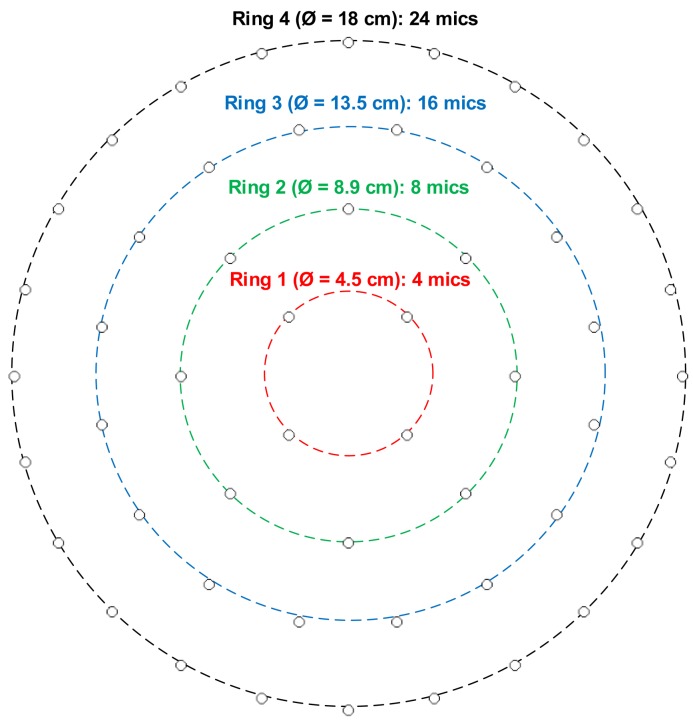
The SoundCompass planar array consists of 52 digital microphones arranged in four concentric rings.

The SoundCompass has a planar microphone array geometry consisting of 52 digital MEMS microphones arranged in four concentric rings (Figure 5). This array is capable of performing spatial sampling of the surrounding sound field by using beamforming techniques. Beamforming focuses the array in one specific direction or orientation, amplifying all sound coming from that direction and suppressing sound coming from other directions. By iteratively steering the focus direction in a 360° sweep, the SoundCompass can measure the directional variations of its surrounding sound field.

Delay-and-Sum Beamforming (DSB) is the simplest of all beamforming techniques [21] and is the one used in the first prototype of the SoundCompass. This section presents a brief introduction to DSB, formally defines an array’s P-PSR and polar directivity, *D_p_*, and uses these parameters to motivate the chosen array geometry depicted in Figure 5.

### 4.1. Delay-and-Sum Beamforming

**Figure 6.**
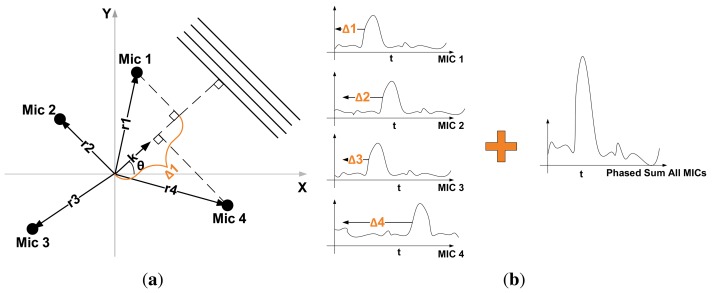
In Delay-and-Sum Beamforming, the output of each microphone in an array is shifted and then added to amplify sound signals arriving from a specific direction. (**a**) In a planar microphone array, the direction vector of a far-field propagating signal with a bearing of *θ* is defined by the unitary vector, *κ*. The time (Δ *_m_*) it takes for this signal to travel from a microphone in the array to the origin is proportional to the projection of the microphone position vector, *r_m_*, on *κ* [22]; (**b**) The Δ *_m_* shift for each microphone is determined by the position of the microphone in the array and the desired focus direction of the array. Signals coming in the same direction as the focus direction will be amplified after the addition of all shifted outputs.

The objective of DSB is to amplify signals coming from a specific direction (array focus) while suppressing signals coming from all other directions. It accomplishes this by delaying the signal output of each microphone by a specific amount of time before adding them all together (Figure 6b), hence its name. The time delay, Δ*_m_*, for a microphone, *m*, in the array is determined by the focus direction, *θ*, and is defined as:

$$\Delta_{m}(\kappa )=\frac{r_{m}\cdot \kappa }{c}$$

where *r_m_* is the position vector of microphone *m*, *κ* is the unitary vector with direction *θ* (Figure 6a) and *c* is the speed of sound.

The DSB’s array output is usually represented in the frequency domain, due to its strong dependence on signal frequency If we define the signal output of each microphone as *S_m_*(*ω*), *ω* = 2*πf* being the angular frequency, then the total output, *O*(*κ*, *ω*), of the array is defined as [22]:

(1) $$O(\kappa ,\omega )=\sum_{m=1}^{M}S_{m}(\omega )e^{-j\omega \Delta m(\kappa )}$$

where *M* is the total amount of microphones in the array. We can further simplify Equation (1) if we consider the case where the array is exposed to a monochromatic acoustic wave:

$$O(\kappa ,\omega )=S_{0}(\omega )\sum_{m=1}^{M}e^{jr_{m}\cdot wn(\kappa_{0}-\kappa )}=S_{0}(\omega )W(wn,\kappa_{0,}\kappa )$$

*W*(*wn*, *κ*_0_, *κ*) is known as the *array pattern*:

(2) $$W(wn,\kappa_{0},\kappa )=\sum_{m=1}^{M}e^{jr_{m}\cdot wn(\kappa_{0}-\kappa )}$$

where *S_0_*(*ω*) is the monochromatic wave, *ωn* is the *wavenumber* ( $wn=\frac{2\pi f}{c}$) of the wave, *κ*_0_ its direction and *κ* the array focus.

The array pattern (Equation (2)) determines the amplification or gain of *S*_0_(*ω*) in the array output. By having *κ*_0_ = *κ*, which is simply focusing the array in the direction of the incoming monochromatic wave, the array pattern reaches its maximum value, *M*. This function describes how well an array can amplify and discriminate signals coming from different directions. It is frequently used to characterize the performance of a sensor array [21,22].

### 4.2. Obtaining the P-SRP

The directional power output of a microphone array, defined here as the polar steered response power (P-SRP), is the array’s directional response to all sound sources present in a sound field. Modeling the sound field at the arrays’ location implies considering multiple broadband sound sources coming from different directions.

An array’s output when exposed to a broadband sound source, *S*, with *n* frequency components incident from direction *κ*_0_ is modeled as:

$$O(\kappa ,S)=S(\omega_{1})W(wn_{1},\kappa_{0},\kappa )+S(\omega_{2})W(wn_{2},\kappa_{0},\kappa )+\ldots +S(\omega_{n})W(wn_{n},\kappa_{0},\kappa )$$

If we assume that the sound field, *ϕ*, where the array is located is composed of different broadband sources coming from different directions, plus some uncorrelated noise, then the output is modeled as:

$$O(\kappa ,\phi )=O(\kappa ,S_{1})+O(\kappa ,S_{2})+\ldots +O(\kappa ,S_{n})+\text{Nois}e_{\text{uncorrelated}}$$

Given that the power of a signal is the square of its absolute value, the array’s power output can be expressed as:

$$P(\kappa ,\phi )={\left|O(\kappa ,\phi )\right|}^{2}$$

We formally define an array’s P-SRP in a sound field, *ϕ*, as the normalized power output:

(3) $$P-\text{SRP}(\theta ,\phi )=\frac{P(\theta ,\phi )}{\underset{\theta \in [0,2\pi ]}{\max}P(\theta ,\phi )}$$

**Figure 7.**
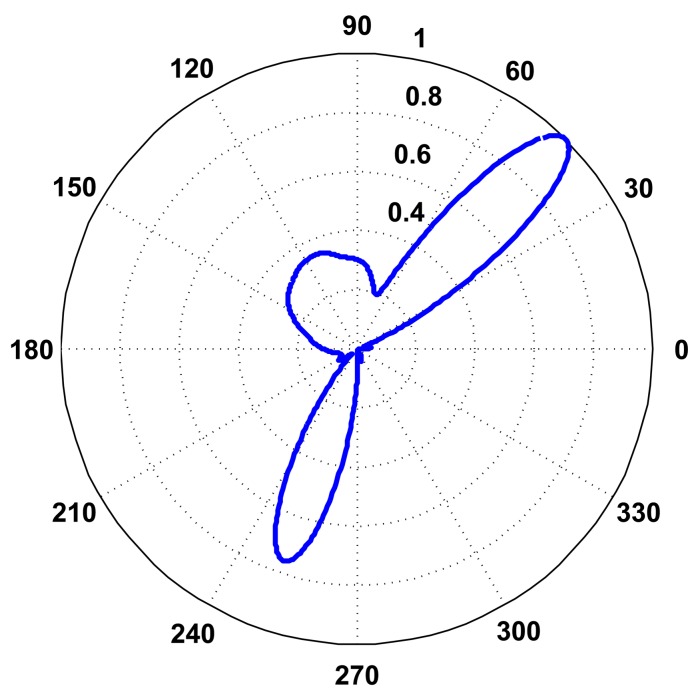
The polar steered response power (P-SRP) output of the SoundCompass when in the presence of three sound sources clearly points to their bearings (45°, 115° and 250°). The frequency spectrum and intensity of each sound source produce differently shaped lobes.

In Equation (3), the direction, *κ*, is replaced with the angle, *θ*, for simplicity, and Figure 7 presents an example of a P-SRP produced by the SoundCompass’ array when exposed to three distinct sound sources, with different bearings, spectra and intensity levels.

The SoundCompass uses a P-SRP to estimate the direction of arrival of nearby sound sources; however, the precision and accuracy of this estimation depends on the directivity of the P-SRP The following subsection defines the concept of directivity and uses it to characterize the overall performance of the SoundCompass as a tool to locate sound sources.

### 4.3. SoundCompass Directivity

**Figure 8.**
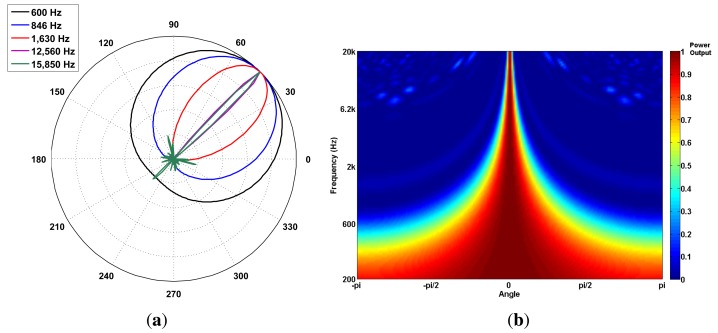
The SoundCompass becomes more directive as the frequency of the measured sound source increases. (**a**) The SoundCompass polar responses at 600; 846; 1,630; 12,560 and 15,850 Hz (all sound sources have the same bearing of 45°) clearly show how the array’s main lobe becomes thinner and, therefore, more directive as the frequency of the sound source increases. (**b**) The waterfall diagram for the SoundCompass shows the power output of the array in all directions for all frequencies. It assumes that the incoming sound wave is at zero degrees. This diagram shows clearly how the directivity of the SoundCompass increases with frequency.

The array polar directivity (*D_p_*) metric determines how effective an array is in discriminating the direction of a sound source. Array directivity is easier to define when considering the scenario of a single sound source. In this scenario, the directivity depends on the P-SRP’s main lobe shape and the capacity of the main lobe to unambiguously point to a specific bearing. Figure 8a shows five different P-SRPs generated by the SoundCompass when exposed to a single sound source (bearing 45°) with five different frequencies. These P-SRPs together with a waterfall diagram (Figure 8b) provide a clear indication of the tendency of the SoundCompass’ array to become more directive as the frequency increases. Array directivity is therefore a key metric for applications, such as sound source localization.

The definition of array directivity for 3D beamforming presented in Taghizadeh *et al.* [13] was adapted here for 2D beamforming and polar coordinates and is as follows:

(4) $$D_{p}(\theta ,\omega )=\frac{\pi P{\left(\theta_{0},\omega \right)}^{2}}{\frac{1}{2}\int_{0}^{2\pi }P{\left(\theta ,\omega \right)}^{2}d\theta }$$

where *P*(*θ*_0_, *ω*) is the maximum output power of the array when focused at the same direction as the incoming sound wave and $\frac{1}{2}\int_{0}^{2\pi }P{\left(\theta ,\omega \right)}^{2}d\theta$ is the output power in all other directions.

**Figure 9.**
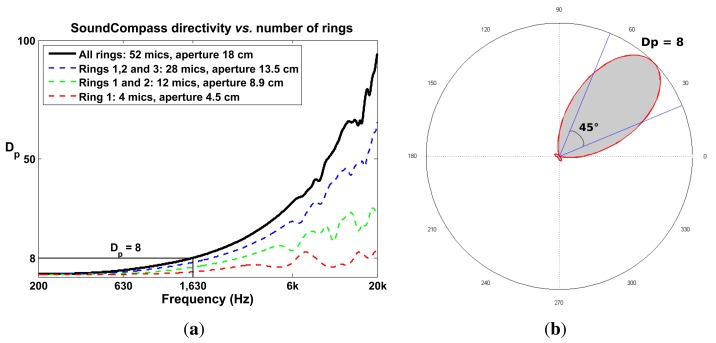
The SoundCompass is able to discriminate a sound source within a half-quadrant (45°) with a frequency of 1,630 Hz or higher. Scaling down the size of the SoundCompass, by removing the outer rings, has a negative impact on the array directivity over all frequencies. (**a**) The SoundCompass polar directivity, *D_p_*, increases with frequency; it surpasses eight after 1,630 Hz. Removing the SoundCompass’ outer rings (see Figure 5) reduces the array aperture and the number of microphones, but also reduces the directivity, *D_p_*. (**b**) The polar directivity, *D_p_*, can be interpreted as the ratio of the area of the unit circle over the area of the P-SRP (gray area). A P-SRP with *D_p_* = 8 has the same area as a 45° sector and is therefore able to pinpoint a direction within a half quadrant.

The top part of Equation (4) can be interpreted as the area of a circle whose radius is the maximum power of the array and the bottom part of the equation the area of the power output. If we normalize these values, *D_p_* becomes an area ratio between the unit circle and a P-SRP (Figure 9b).

The value of *D_p_* for the SoundCompass reaches eight at 1,630 Hz (Figure 9a). A value of eight implies that the SoundCompass’s main lobe is eight times smaller than the unit circle and, therefore, has a comparable surface area as a 45° sector (Figure 9b). The main lobe can then confidently estimate the bearing of a sound source within half a quadrant. For practical reasons, we take this location resolution as the minimum for sound source localization (see Section 5). After 1,630 Hz, the SoundCompass generates a thin main lobe with almost no side lobes. This behavior is desired for sound source localization and sound mapping applications.

### 4.4. Motivating the Array Geometry

We chose a circular array geometry to maintain the array’s P-SRP, and also *D_p_*, independent of orientation. Our array’s radial symmetry removes the existence of good or bad array orientations typically found in linear arrays; a similar argument is presented in [16] to justify the use of circular arrays.

*D_p_* increases as the total diameter of a circular array increases (Figure 9a); therefore, as for most sensor array applications, the bigger, the better. Nevertheless, the constraints typically found in WSN applications limit the size of a sensor node. A large sensor node is expensive, difficult to deploy and cumbersome to handle. A sensor node with a 20-cm diameter PCB size was a sensible compromise between size and cost; it is still fairly cheap to have PCBs of this size produced in low quantities, while large enough to build an array with good directivity in the low frequency ranges.

The total number of microphones has a direct effect on the array gain; adding more microphones to the array increases the array’s output signal-to-noise ratio (SNR). Additionally, as microphones are positioned closer together, the directivity, *D_p_*, increases in the high frequency ranges [21,22]. However, more microphones means higher power consumption, a critical parameter in a WSN. Power consumption optimization is not analyzed in this article; therefore, to safely demonstrate our proposed localization technique, we opted to use 52 microphones. This amount of microphones is about the maximum that can easily be routed and interfaced on a double layer PCB of a 20-cm diameter and pushes the limits of the FPGA used to interface and implement the DSB algorithm.

## 5. Distributed Sensing

The SoundCompass is designed to work as part of a group of geographically distributed sensors in a WSN. In this WSN, each node is equipped with a SoundCompass and radio frequency (RF) communication capabilities to relay to a sink the total SPL and directionality information of its surrounding sound field. At the WSN’s sink, the collected data is combined or *fused* to acquire a global view (for example, a noise map) of the covered geographical area.

A WSN equipped with a SoundCompass on each node can locate multiple sound sources within its coverage area by fusing all its P-SRPs. This fusion generates a *probability map* of the estimated location of sound sources within this area. The following subsections describe the probability map fusion technique together with simulations that evaluate its performance and limitations.

### 5.1. Probability Maps

Probability Map is a data fusion technique for locating sound sources using the SoundCompass and a wireless sensor network (WSN). The SoundCompass, in the presence of one or several sufficiently loud sound sources, outputs a P-SRP with the directional information of the corresponding sound sources (Figure 7).

By combining the power plot outputs of all nodes in a 2D field, it is possible to determine the location of all sound sources with varying degrees of accuracy. The localization accuracy will depend on several factors:

- Node density: Specifies how many nodes cover the field and how far apart they are.
- Angle resolution: Specifies the resolution of the P-SRP or how many measurements per 360° sweep the array performs.
- Sound source frequency spectrum: Specifies the frequency decomposition of the sound source.
- Number of sound sources: Specifies the number of detectable sound sources (above the noise floor of the array’s microphones) present in the field.

The group of techniques used to combine the data generated by several sensors is called data fusion, and in this case, it implies the superposition of each P-SRP to create a probability map of sound source locations.

**Figure 10.**
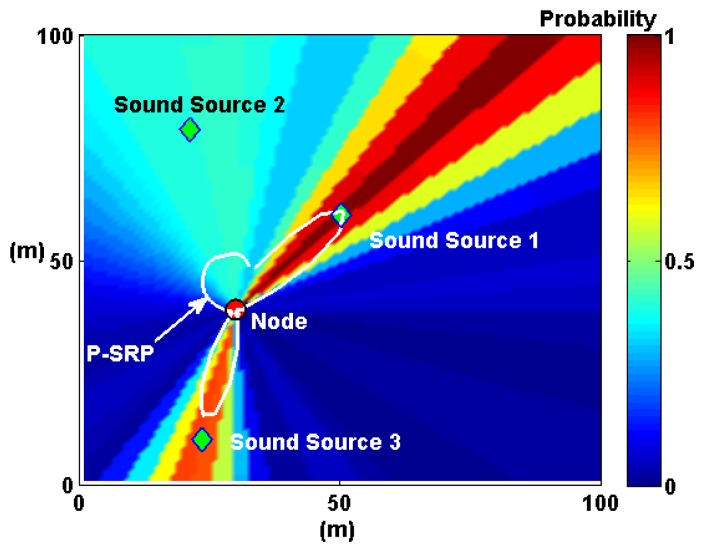
P-SRP illumination is visualized here as the radial propagation of the P-SRP values over the entire map area. The same P-SRP presented in Figure 7 is shown here with the SoundCompass node at its center and three surrounding sound sources in a 100 m × 100 m probability map. The sectors containing the three sound sources are *illuminated* with higher probability values.

Expanding on the microphone array fusion techniques developed by Aarabi and DiBiase to detect speakers in indoor environments [5,10], we propose P-SRP superposition. This method uses the P-SRP generated by each node to *illuminate* sectors of a map representing the deployment area of the WSN. P-SRP map illumination is defined here as the radial propagation of the P-SRP values over the entire map area (Figure 10). It is formally defined as follows:

Let *m_n_* be a matrix representing the geographical area where a WSN containing node *n* is deployed. The dimensions of *m_n_* are determined by the spatial size of the entire deployment and the desired resolution (for example, a 1-m resolution map of a deployment covering an area of 10,000 m^2^ will produce a 100 × 100 map matrix).

The angle function, *β*(*a*, *n*), returns the angle between node *n* and a point, *a*, in *m_n_* and is defined as:

$$\beta (a,n)=\arctan(\frac{a_{y}-n_{y}}{a_{x}-n_{x}})$$

The matrix *m_n_*, with dimensions *i*, *j*, is then defined as:

(5) $$m_{n}=\left[\begin{array}{ccc} P-SRP_{n}(\beta (a_{1,1},n),\phi ) & \cdots & P-SRP_{n}(\beta (a_{1,j},n),\phi )\\ \vdots & \ddots & \vdots \\ P-SRP_{n}(\beta (a_{i,1},n),\phi ) & \cdots & P-SRP_{n}(\beta (a_{i,j},n),\phi ) \end{array}\right]$$

where *P-SRP_n_*(*θ*, *ϕ*) is the P-SRP produced by node *n* (Equation (3)). Figure 10 shows an example of P-SRP illumination by plotting matrix *m_n_* in a pseudocolor plot. In this plot, the sectors where the sound sources are found are illuminated with higher probability values.

The next step to generate a complete probability map is P-SRP superposition. In this step, the maps generated by each node are added together and normalized to obtain the total raw probability map, *M*. For a total number of nodes, *N*, *M* is defined as:

(6) $$M=\frac{\overset{N}{\underset{n=1}{\text{∑}}}m_{n}}{N}$$

**Figure 11.**
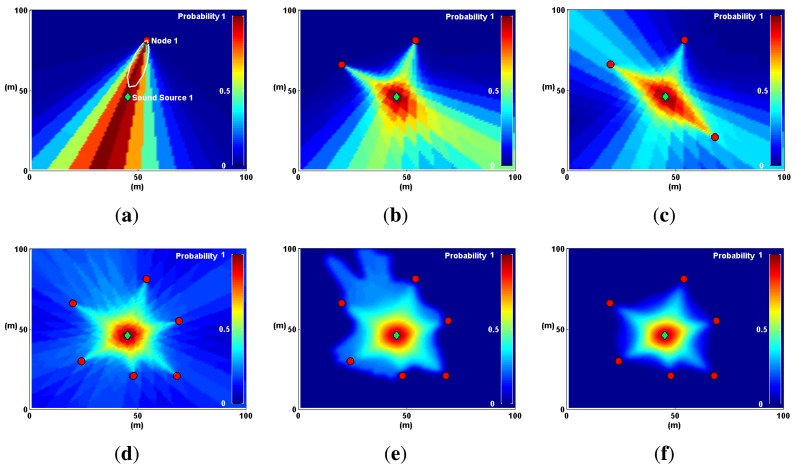
A single sound source and several SoundCompass nodes are simulated here in a 100 m × 100 m field to illustrate the Probability Map technique. (**a**) One node illuminates the sector where the sound source is found with high probability values. (**b**) With two nodes, the map superposition homes-in on the real sound source location. (**c**) Adding extra nodes increases the positioning accuracy. (**d**) The positioning accuracy increases with more nodes, but so does the map noise. (**e**) Image filtering removes map noise to allow a peak finding algorithm to locate the sound source. (**f**) A distance degradation optimization reduces map noise from areas where no sound sources are detected.

As the number of nodes increases, the probability map, *M*, produces *hot spots*, where sound sources are most likely to be found. This process is illustrated in Figure 11.

The sequence in Figure 11 illustrates the main process behind the technique; however, a couple of optimization steps need to be performed before using a probability map to locate sound sources. The first step, evidenced in Figure 11e, is image filtering, where map noise is smoothed out using a low-pass Gaussian filter. The second step is distance degradation. The *illumination* produced by each P-SRP must be degraded with distance (shown in Figure 11f) to further diminish map noise.

To evaluate the limits of the probability maps technique and to determine the optimal firmware configuration for the SoundCompass when working in a distributed environment, we performed a set of simulations. The following sections explain in further detail the optimization techniques used and the results of the simulations.

#### 5.1.1. Gaussian Image Filter

A probability map becomes *hot* in areas where sound sources are most likely to be found, and to measure the localization accuracy of this map, it is necessary to automate the process of finding these *hot* or local maxima areas. A common method to accomplish this is a peak finding algorithm; such an algorithm traverses the map looking for local maxima points: specific points where all its neighbors are of a smaller value. However, this algorithm tends to output a significant amount of false positives, due to small *wrinkles* in the map surface (map noise). Smoothing the map surface is therefore necessary to improve the accuracy of a probability map.

**Figure 12.**
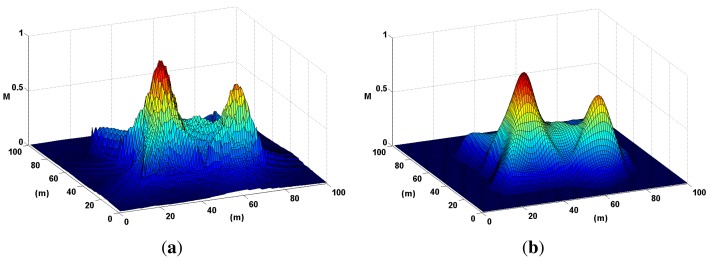
Noise is smoothed out of a probability map using a low-pass Gaussian filter. This step is mandatory to accurately locate local maxima or peaks in the map. (**a**) A probability map of two sound sources is presented here in three dimensions to highlight the effects of noise. (**b**) The probability map in (a) is smoothed out here using a Gaussian filter.

In [11], the authors propose spatial smoothing using image filtering techniques (for example, using a low-pass Gaussian filter) to remove noise from microphone SRP-PHAT data fusion. A low-pass two-dimensional Gaussian filter transfer function is defined as:

$$H(r_{x},r_{y})=\frac{1}{2\pi \sigma^{2}}e^{-\frac{r_{x}^{2}+r_{y}^{2}}{2\sigma^{2}}}$$

where *σ* and the kernel size are adjustable values that can be changed depending on the scale of the map. Figure 12 illustrates the results of applying the filter, *H*(*r_x_*, *r_y_*), in a probability map, *M*, where two sound sources are present.

#### 5.1.2. Distance Degradation

Distance degradation is a method that estimates *a priori* the position of sound sources before the computation of the probability map. It assigns a lower probability to areas of the map where a node is less likely to find a sound source. This method is akin to the Spatial Observability Function (SOF) defined in [10] to locate sound sources (voice) in an indoor environment. In SOF, areas in the room that were not physically accessible by a microphone array (for example, behind a wall) were given low probability.

**Figure 13.**
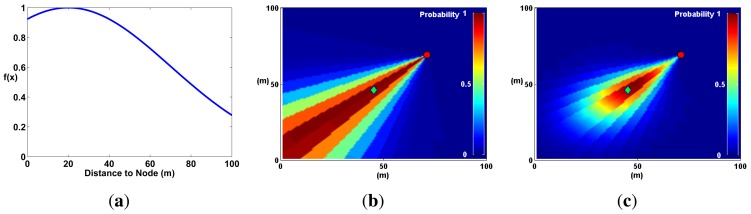
Applying the Gaussian function in (a) to a map *m_n_* (b) produces a degradation of the probability with respect to the distance to the node (c). (**a**) A Gaussian curve representing the expected distribution of node to sound source distances. The expected value, μ, in this case is 20 m. (**b**) Without distance degradation, the probability keeps constant in relation to the distance from the node. (**c**) Multiplying the probability in each sector with the Gaussian function in (a) generates a probability map where probabilities decay with distance.

In an open field, SOF can be generalized by assuming that sound sources are, on average, at a certain distance from the microphone array. At that distance, the probability to find a sound source is highest, and everywhere else, the probability decays using a Gaussian distribution (Figure 13). This optimization reflects the fact that a SoundCompass node has a finite distance range to detect sound sources.

The effects of distance degradation help eliminate noise coming from unwanted or uninteresting sound sources (evident in Figure 11f). Moreover, Aarabi demonstrated that SOF improved localization accuracy by a few centimeters in an indoor environment [10]. Gaussian distance degradation has two adjustable parameters: average and standard deviation. These values can be used to fine-tune the Probability Map technique to take into account different urban situations (e.g., street and building layout, area coverage and typical noise levels). It was used in all the simulations described in the next subsection.

### 5.2. Simulations

**Figure 14.**
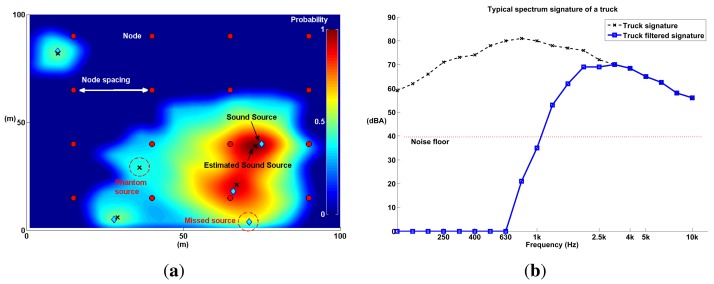
Several simulations of a wireless sensor network (WSN) equipped with a SoundCompass on each node on a 100 m × 100 m open field were performed. The spectrum signature of a heavy truck was used as a test sound source in most simulations. Several variables were iterated (node spacing, number of sound sources, angle resolution, sound source frequency, *etc.*) to measure the performance of the localization technique. (**a**) A node grid spaced 25 m apart accurately detects four out of five sound sources. The map shows two kinds of map errors: a *miss* (a not detected sound source or false negative) and a *phantom* (a false positive). (**b**) The typical frequency spectrum of a heavy truck [23] has a peak at around 1 kHz. However, to improve the localization accuracy, frequencies below 1,630 Hz must be filtered out. The blue line is the result of filtering the truck signature with a high pass filter, and the red line represents the simulated noise floor in all microphone arrays.

We performed several simulations to determine the limits of the probability mapping technique in terms of the following four parameters (see Figure 14a): node spacing, angle resolution, number of sound sources and sound source frequency spectrum. Node spacing and angle resolution are directly related to the deployment of a WSN, while the number of sound sources and the frequency spectrum are tied to the physical limitations of the technique.

Node spacing—the average distance between nodes—determines the density of the network; a small node spacing implies a high density WSN, and this, in turn, implies more costs. On the other hand, angle resolution determines the amount of computing resources utilized by the SoundCompass. Higher angle resolutions require higher sampling rates and, therefore, higher energy consumption, a critical parameter in a WSN. How does node spacing and angle resolution affect the accuracy and quality of a probability map? What are their optimal values?

The number of sound sources and the frequency spectrum are tied to the limitations of the technique. What is the maximum amount of simultaneous sound sources that can be detected in a typical distributed SoundCompass deployment? How does the spectrum of a sound source affect the localization accuracy?

We designed a set of simulations to find some insight into possible answers to the questions posed in this section. The setup and configuration of all simulations is presented in the following section.

#### 5.2.1. Simulation Setup

All simulations were performed in a MATLAB environment, where a virtual open field of 100 m × 100 m was simulated (10,000 m^2^). This area is approximately the area of a busy street intersection—one of the settings where we envision to deploy the SoundCompass. To simplify all simulations, we assumed that there are no reverberations, and sound propagation follows an open field spherical radiation model:

(7) $$\text{SPL}(d_{s})=SWL_{s}-20\log(d_{s})+K$$

where *SPL*(*d_s_*) is the SPL at a distance, *d_s_*, from a sound source, *SWL_S_* is the sound watts level (*i.e.*, sound power level) of the source and *K* is a propagation constant. One of the implications of Equation (7) is that the value of SPL drops 6 dB when doubling the distance [24].

The simulations proceed as follows: A node grid—each node containing a SoundCompass—is placed within the open field (see Figure 14a). Each node in this grid measures the virtual sound field generated by the simulation to produce a P-SRP that is then used to produce a probability map.

With the exception of the frequency spectrum simulations, all sound sources had the typical frequency spectrum of a heavy truck (see Figure 14b) to further approximate the environment of a busy urban intersection. For the frequency spectrum simulations, the frequency of sound sources was iterated, using 1/3 octave bands resolution. In each simulation iteration, all sound sources were placed randomly within the field. Additionally, a noise floor was simulated in every node. The value of the noise floor was determined from the specification of the MEMS microphones used in the SoundCompass. As sound propagated and reached a node, if the power of the SPL was below the noise floor of the SoundCompass, random noise was generated as the node P-SRP output.

Three test parameters—node spacing, angle resolution and the number of sound sources—iterated from 10 m (81 nodes) to 50 m (four nodes), eight to 64 resolution and one to 10 sources, respectively. Each iteration was a combination of these three parameters. Additionally, each iteration was repeated 15 times and each time all sound sources were placed randomly within the map.

Time was not simulated; therefore, sound sources were static, and their frequency spectrum did not change. The communications aspects—routing, time synchronization, interference, and so forth—of the WSN were not simulated.

Each iteration produced a probability map. This map was optimized using Gaussian degradation and a low-pass Gaussian filter. The Gaussian degradation expected value (μ) was set to the *node spacing* value of the iteration to optimize the localization of sound sources within the node grid. For the low-pass Gaussian filter, we set *σ* = 3 and used a kernel size of 5 × 5 pixels.

Each produced probability map was evaluated using three metrics: localization error, average number of misses and average number of phantoms (see Figure 14a). These metrics quantify how well the probability map locates sound sources as a function of the test parameters. The localization error is the distance between the estimated sound source and the real sound source. A miss is a sound source that was not located by the probability map (a false negative), and a phantom is a located sound source that does not correspond to any real sound source (a false positive). These three parameters were averaged, over 15 repetitions, for each iteration. The results are presented in the next subsection.

#### 5.2.2. Simulation Results

**Figure 15.**
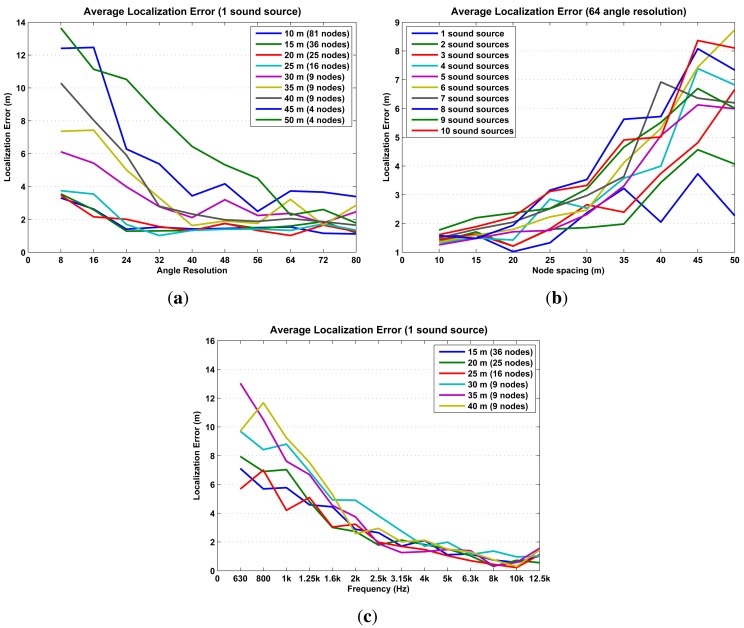
The average localization error is clearly correlated with the angle resolution, node spacing, and with the spectrum of the sound source. (**a**) The average localization error of one source is below 4 m, with an angle resolution of 64 or more. For angle resolutions larger than 64, there is almost no improvement on error localization either by decreasing the node separation or increasing the angle resolution. (**b**) When using an angle resolution of 64, the average localization error of one to 10 sound sources is consistently below 4 m when the node separation is 30 m or less. (**c**) The average localization error of one source decreases as the frequency of the sound source increases. It drops below 4 m from 2,500 Hz on, where it exhibits almost no correlation with node spacing.

**Figure 16.**
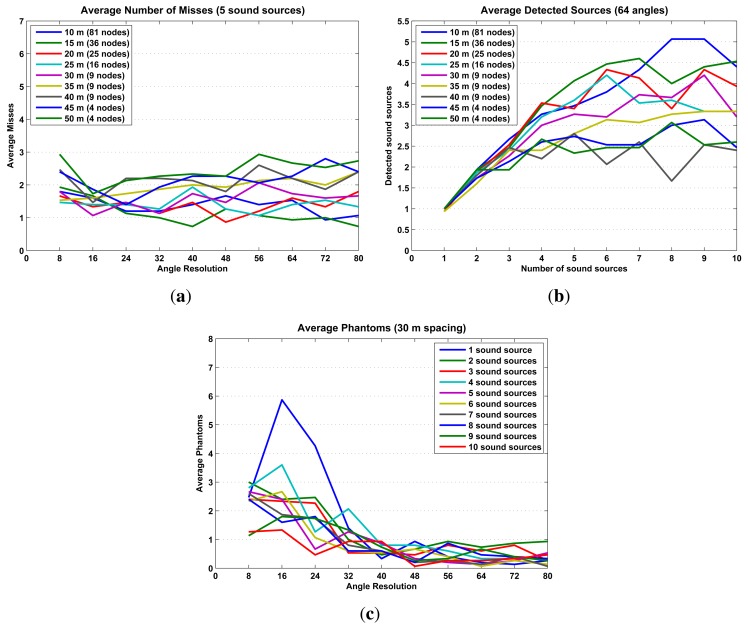
These three graphs illustrate how false negatives (reflected in the number of misses and detected sound sources) and false positives (known in this domain as phantoms) relate to other map parameters. (**a**) When five sound sources are present in the simulation, the average number of misses hovers between one and three, regardless of the angle resolution or node spacing. (**b**) The average detected number of sound sources reaches a maximum of five as the number of present sound sources increases. This implies an inherent limit of around five detectable sound sources in a 100 m × 100 m field. (**c**) A good angle resolution helps to reduce the number of false positives; for angle resolutions larger than 40, the average number of phantoms stabilizes at a value of one or less.

The simulations results are presented in Figures 15 and 16 in terms of localization error, misses and phantoms. Additionally, Figure 16b shows results in terms *of detected sound sources*, which are the total number of sound sources in an iteration minus the number of misses.

The simulation results already establish some initial limitations and optimal configurations for using probability maps to locate sound sources. The optimal configuration, high precision/low cost, is to configure the SoundCompass with a 64 angle resolution (Figure 15a) and place all nodes at a spacing distance of 30 m or less (Figure 15b). This setup will be able to accurately locate up to five sound sources (Figure 16b) with an average localization accuracy of 2.3 m or less (from Figure 15a and b, 30-m spacing, 64 angle resolution).

Additionally, as the frequency spectrum of a sound source shifts to the lower frequencies, the localization error increases rapidly (Figure 15c). This is a consequence of the poor directionality of the SoundCompass’ P-SRP in the low frequencies. To compensate for this limitation, only frequencies above 1,630 Hz must be used to generate probability maps; lower frequencies should be filtered out from the SoundCompass’ P-SRP output. While this limitation precludes the localization of sound sources with frequencies below 1,630 Hz, most targeted sound sources—cars, trucks, trams, and so on—have strong frequency components above 1 kHz [23].

## 6. Results and Discussion

To validate our SoundCompass, we submitted our prototype to three separate tests: a directionality test, an angular resolution test and a localization experiment.

- Directionality: The main function of the compass is to determine the angle of arrival (AoA) of the sound emitted by a sound source. We tested the directionality of the array to see how precise the AoA reported by the SoundCompass actually is. The frequency of the source influences the results of the DSB technique we use to locate the source; therefore, the directionality was measured in relation to the frequency.
- Angular Resolution: A second test determines the ability of the SoundCompass to distinguish two sound sources that are positioned close to each other. The angular resolution should not to be confused with the previously mentioned angle resolution.
- Localization Experiment: A third test was the reproduction of a real-life use case of the SoundCompass. In this test, several sound sources are being located by the combination or fusion of the measurements of several SoundCompasses.

All tests were conducted in an anechoic room to simulate an open field environment. Since the complete FPGA implementation was not finished at the time of testing, the FFT calculations were offloaded to a computer running MATLAB; all other processing steps were conducted on the SoundCompass itself. All tests are representative for the finished prototype, since there are no hardware differences in the microphone array itself.

### 6.1. Directionality

**Figure 17.**
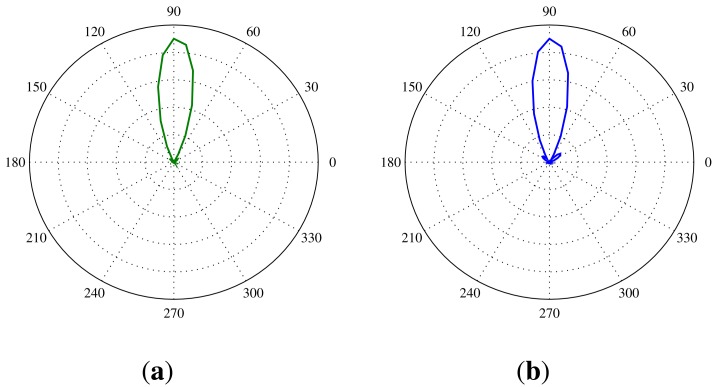
Power plots produced by the SoundCompass as a result of a sound source positioned 90 cm from the array at a 90° angle and producing a tone of 3,150 Hz. (**a**) The power plot produced by our simulation of the SoundCompass; (**b**) The power plot produced by the SoundCompass.

The directionality of the SoundCompass will determine how precise it can determine the AoA of a sound source. This translates into a test setup in which one sound source is placed at a known angle according to the SoundCompass. Then, several measurements are taken by the SoundCompass with the sound source each time producing a tone at a different frequency. We chose to use the center frequency of octave bands ranging from 500 Hz to 20 kHz for this test, since the half quadrant resolution of the SoundCompass is 1,630 Hz. After doing a measurement for each octave band, the SoundCompass produces a P-SRP for that frequency or band; e.g., Figure 17b shows the P-SRP for a measurement of a source at a 90° angle producing a constant tone of 3,150 Hz.

**Figure 18.**
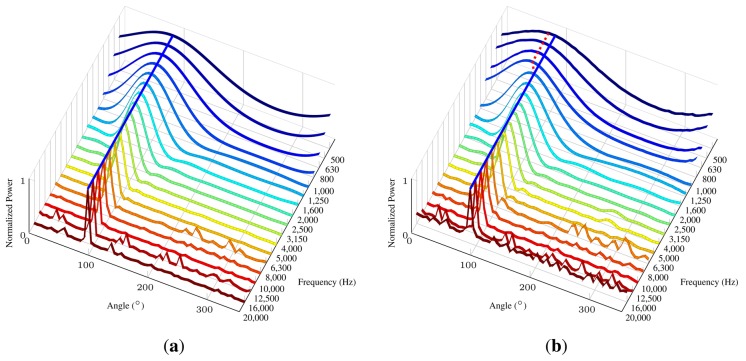
Theoretical and experimental directionality graphs for the SoundCompass with a fixed source at 90° with a varying frequency from 500 till 20 kHz. The Blue line marks the 90° angle. The red dotted line marks the directionality obtained by the experiment. (**a**) The directionality graph for the SoundCompass according to our theoretical model; (**b**) the experimental directionality graph for the SoundCompass. Directionality in the lower frequencies deviates from the theoretical model.

To easily determine the directionality of the SoundCompass for every frequency in the chosen spectrum of interest, we can now plot a graph in which all frequency bands with the power for every angle is compared. Figure 18 shows a stretched out P-SRP (64 angle resolution) for each frequency band. The bearing of the sound source is 90 degrees.

On this graph, we can now see how the highest peak changes according to the frequency. This peak corresponds to the angle where the SoundCompass thinks the sound source is located. Figure 18a pictures the directionality graph of the SoundCompass with a source at a 90° angle with the data of our theoretical model and simulation of the SoundCompass. We consider this graph to be the ideal directionality. The blue line in the graph emphasizes the 90° angle, and the power plot in every frequency perfectly peaks at that angle. Figure 18b shows the same ideal blue line at 90°, but it is noticeable that the maxima in the power plot of the lower frequencies are slightly off, which is marked by the red dotted line. This deviation in the lower frequencies is a result of the smaller size of the SoundCompasses’ array and an imperfect open field environment. The deviation is in the order of 10° and stays constant during one test setup, but can switch polarity over multiple different setups. When comparing the side lobes of our theoretical model with the ones resulting from real-life tests, a very close resemblance can be seen, with some extra noise in the tests resulting from reflections, due to an imperfect anechoic environment. There is also a clearly higher noise value in the 20 kHz band compared to the theoretical model. This is most likely due to the non-ideal response of the microphones at this frequency. Since this frequency is at the upper limit of the audible spectrum and is not perceived by most humans, we disregard the added noise in this band. Because of the radial symmetry of the SoundCompass, we suppose that these measurements are uniform over the complete angular range of the sensor.

It is important to note that at this stage of our research, we have not taken into consideration the effects of reverberations. Reverberations are a major concern in indoor environments, where it is commonly accepted that it negatively affects the performance of localization algorithms [10,11,16]. However, by carefully studying an indoor environment, reverberations can be used to increase the accuracy of localization algorithms, as shown in [25]. Moreover, our main application domain, environmental noise monitoring, is usually outdoors, where reverberations, while not negligible, are of lesser concern.

### 6.2. Angular Resolution

**Figure 19.**
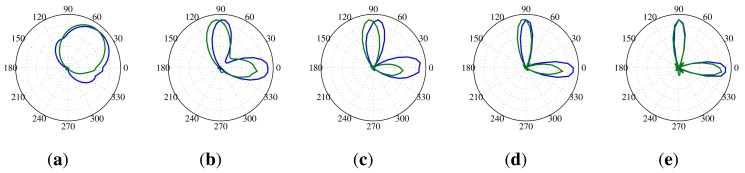
These P-SRPs are the result of a test setup with two sources positioned 70 cm from the SoundCompass at an angle of 0° and 90°. The blue line represents the power plot produced by the SoundCompass, and the green line represents the power plot according to our theoretical model. (**a**) Two 1,250 Hz sources at 90° and 0°; (**b**) two 2,000 Hz sources at 90° and 0°; (**c**) two 2,500 Hz sources at 90° and 0°; (**d**) two 3,150 Hz sources at 90° and 0°; (**e**) two 4,000 Hz sources at 90° and 0°.

**Figure 20.**
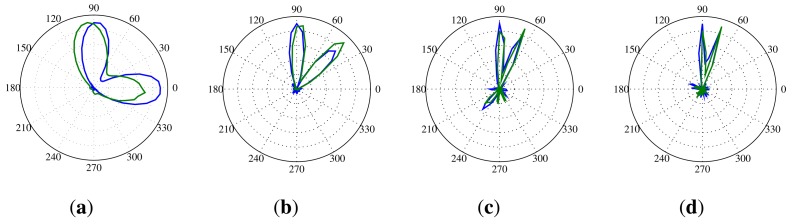
These P-SRPs show the frequency limits at which two equal sources become distinguishable for four different angles. The blue line represents the power plot produced by the SoundCompass, and the green line represents the power plot according to our theoretical model. (**a**) Two 2,000 Hz sources at 90° and 0°; (**b**) two 4,000 Hz sources at 90° and 45°; (**c**) two 8,000 Hz sources at 90° and 67.5°; (**d**) two 10,000 Hz sources at 90° and 37.1°.

The angular resolution determines how well the SoundCompass can distinguish two different noise sources. This translates for the SoundCompass into the minimum angle at which it can distinguish two sources. To measure this angle, we placed two identical sources 70 cm from the SoundCompass and kept one source stationary while, at every step, moving the other source closer. The width of the main lobe in the P-SRP produced by the SoundCompass depends on the frequency of the sound source, and because of that, the angular resolution is correlated with the frequency of the sound sources. Figure 19 shows the P-SRPs of two sources, one at an angle of 0° and one at 90°, for frequency bands varying from 1,250 Hz until 4,000 Hz. In these P-SRPs, the green graph again represents the simulated power calculated by our theoretical model, and the blue graph represents the measured power by the SoundCompass. The graphs show that two sources that are 90° apart can be distinguished if they have a frequency higher than 2,000 Hz. Similar frequencies for other angles are pictured in Figure 20.

**Figure 21.**
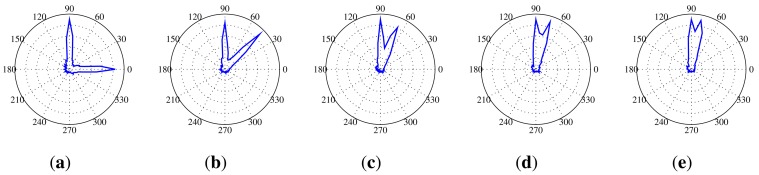
These P-SRPs are the result of a test setup with two sources producing pink noise positioned 70 cm from the SoundCompass, one at an angle of 90° and the other one varying from 0° until the smallest possible angle. (**a**) Two sources producing pink noise at 90° and 0°; (**b**) two sources producing pink noise at 90° and 45°; (**c**) two sources producing pink noise at 90° and 67.5°; (**d**) two sources producing pink noise at 90° and 73.1°; (**e**) two sources producing pink noise at 90° and the smallest measurable angle by the SoundCompass.

Because the noise sources measured by the SoundCompass will rarely be monochromatic, we repeated this test with two pink noise sources. Pink noise is random noise having equal energy per octave and, so, having more low-frequency components than white noise. The first source was again kept stationary at 90° and 70 cm from the SoundCompass, while the second source started at 0° and was moved closer towards 90° with every measurement. Since the angle resolution of the SoundCompass is $\frac{360{}^{\circ}}{64}=5.625{}^{\circ}$, the minimum angular resolution of the SoundCompass would be 2 × 5.625° = 11.25°, because we need a minimum of three neighboring points in the power plot to distinguish two separate maxima. Figure 21 shows the power plots from this test with the angle between the two pink noise sources varying from 90° to the minimum measurable angle. These graphs clearly show that even at the minimum angle, the two pink noise sources are still clearly distinguishable. This is mainly due to the high frequency components present in pink noise, resulting in a very narrow high peak in the P-SRP.

### 6.3. Localization Experiment

**Figure 22.**
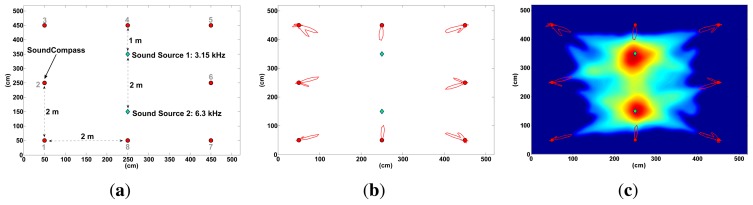
We placed two monochromatic sound sources at the center of an anechoic chamber and placed our SoundCompass in eight different positions within this chamber (a). The polar graphs generated at each position (b) were used as the input in our data fusion algorithm to obtain a probability map (c). The localization errors for Sound Source 1 and Sound Source 2 were 10 cm and 2.2 cm, respectively. (**a**) Anechoic chamber setup: two sound sources and one SoundCompass in eight different positions; (**b**) the resulting polar graphs are displayed here with respect to their positions; (**c**) the generated probability map located the two sound sources with an accuracy of 10 cm and 2.2 cm.

To test the distributed sound localization capabilities of the SoundCompass in an open field, we placed our device in an anechoic chamber (5 m × 5 m) together with two monochromatic sound sources of equal power, but different frequencies (Figure 22a). The SoundCompass measured the directivity of the sound field in eight different positions within the chamber. These measurements—polar graphs with 64 angle resolution—were used as inputs to the data fusion algorithm described in Section 5.1. The resulting probability map (Figure 22c) was able to locate Sound Source 1 with an error of 10 cm and Sound Source 2 with an error of 2.2 cm.

A complete simulation of the experiment in Figure 22 yielded a localization error of 6.9 cm for Sound Source 1 and 2.4 cm for Sound Source 2. The small difference between the simulated localization error and the localization error obtained in the anechoic chamber can be explained by the small *placing* error (±2 cm) of the SoundCompass and the two sound sources in their respective positions within the chamber.

## 7. Conclusions and Future Work

We presented the SoundCompass, an acoustic sensor able to measure sound intensity and directionality. The sensor is able to measure a local SPL value and produce a P-SRP. The P-SRP points, like a compass, to the sound sources surrounding the sensor. It can be used in a standalone application for sound source detection and comparison, but is optimized for its distributed measurement setup. In this scenario, multiple SoundCompasses are deployed in an area of interest and are networked via a WSN. This enables them to take simultaneous measurements that, when fused together, help locate multiple sound sources present within the coverage area of the WSN.

We presented the sensor architecture, which is based on a small circular microphone array driven by an FPGA, and characterized the performance of the array. For the eventual goal of producing sound maps with the SoundCompass and its supporting WSN, we introduced a data fusion technique called Probability Maps. This technique uses P-SRP superposition to determine possible sound source locations and to create a probability map of these locations. We applied this technique on our SoundCompass and concluded that a network with nodes spaced at a distance of 30 m or less with sensors configured with a 64 angle resolution can locate up to five sound sources with an average accuracy of 2.3 m (100 m by 100 m field).

To conclude the tests and to validate the complete system, we performed a live test in an anechoic chamber (5 m × 5 m) with two sound sources surrounded by eight SoundCompasses. The resulting probability map, which was generated by combining the measurement data of each individual SoundCompass, located the two sources with an accuracy of 10 cm and 2.2 cm. This result confirms that the SoundCompass as a sensor and the Probability Map fusion technique can accurately locate sound sources in an open space.

After testing our SoundCompass prototype, it behaved as predicted by our theoretical models, and our directionality experiments demonstrated that it can easily discriminate the bearing of two broadband sound sources. However, when these sound sources contained strong frequency components below 2 kHz, our prototype was not able to properly differentiate them. This behavior—a poor directionality response in frequencies below 2 kHz—is a consequence of the relatively small array aperture (18 cm) and the DSB algorithm. Our future prototypes will implement more advanced beamforming algorithms to help compensate for these limitations.

During our simulations and experiments, we noticed that the amount of microphones currently used on the SoundCompass can probably be reduced, while still resulting in almost equally good results. Because of this, one of our next steps will be to construct new prototypes with fewer microphones to further reduce the cost of the sensor and to determine where the exact threshold lies between the amount of microphones and the needed accuracy and resolution.

One of the still untouched parts of the distributed measurement network consisting of SoundCompasses is the WSN part. This WSN will fulfill the important task of initiating measurements and collecting all the measurement values of the different sensors to a central collection point. These measurements need to be initiated at exactly the same time, so a very strict time syncing protocol will be needed. Further research on powering a SoundCompass network via miniature energy harvesting devices is also being conducted.

The main contribution of the SoundCompass is the combination of two technologies—MEMS-based microphone arrays and SRP-based data fusion—to locate sound sources in a new application domain: environmental sensor networks. In this domain, our final aim is to use the SoundCompass to improve the generation of noise maps. By taking into account the positions of located sound sources in the interpolation process, the resulting noise map provides a more accurate representation of the environmental reality with clearly discernible noise pollution sources. We believe that the SoundCompass is a new sensor that will enable sound engineers and policy makers to assess, map and fight noise pollution in a more effective way. When the sensor is being used in its distributed measurement model, it can deliver sound pollution data with a higher precision than the currently used pollution assessment tools. These more accurate and up-to-date measurements will enable a clearer representation for the general public to asses the pollution levels around them and, thus, enable them to make better decisions to avoid or fight sound pollution. The SoundCompass as an individual sensor will also enable new audio analysis devices with a wider scope than only pollution monitoring. In the future, we see the SoundCompass incorporated as a sensor in sound cameras, location tracking devices, multi-camera setups and even consumer electronics.
